# Regression Networks for Neurophysiological Indicator Evaluation in Practicing Motor Imagery Tasks

**DOI:** 10.3390/brainsci10100707

**Published:** 2020-10-04

**Authors:** Luisa Velasquez-Martinez, Julian Caicedo-Acosta, Carlos Acosta-Medina, Andres Alvarez-Meza, German Castellanos-Dominguez

**Affiliations:** Signal Processing and Recognition Group, Universidad Nacional de Colombia, Manizales 170004, Colombia; juccaicedoac@unal.edu.co (J.C.-A.); cdacostam@unal.edu.co (C.A.-M.); amalvarezme@unal.edu.co (A.A.-M.); cgcastellanosd@unal.edu.co (G.C.-D.)

**Keywords:** sensorimotor rhythm, event-related de/synchronization, brain-computer inefficiency, regression networks

## Abstract

Motor Imagery (MI) promotes motor learning in activities, like developing professional motor skills, sports gestures, and patient rehabilitation. However, up to 30% of users may not develop enough coordination skills after training sessions because of inter and intra-subject variability. Here, we develop a data-driven estimator, termed Deep Regression Network (DRN), which jointly extracts and performs the regression analysis in order to assess the efficiency of the individual brain networks in practicing MI tasks. The proposed double-stage estimator initially learns a pool of deep patterns, extracted from the input data, in order to feed a neural regression model, allowing for infering the distinctiveness between subject assemblies having similar variability. The results, which were obtained on real-world MI data, prove that the DRN estimator fosters pre-training neural desynchronization and initial training synchronization to predict the bi-class accuracy response, thus providing a better understanding of the Brain–Computer Interface inefficiency of subjects.

## 1. Introduction

Motor Imagery (MI) is understood as the dynamic cognitive ability to generate mental movements without performing them. This mental process triggers the activation of the neurocognitive mechanisms that underlie the planning of voluntary movements resembling how the action is executed in a real way [1]. Currently, MI has been postulated as a reliable tool to promote motor learning in all of its phases in activities, like the development of professional motor skills learning [2], improvement of sports gestures [3], and enhancement of skilled performance in the patient rehabilitation [4,5], among others. In these applications, the Media and Information Literacy methodology proposed by UNESCO includes many competencies that are vital for people to be effectively engaged in human development [6]. Electroencephalography (EEG) signals are broadly accepted to implement MI systems due to their noninvasive nature, portability, and cost-efficiency. However, the most common limitations for decoding neural responses are related to the inter and intra-subject variability that leads to non-stationary, nonlinear, and a poor signal-to-noise ratio of EEG signals. All of these factors, along with frequently used small datasets, decrease the performance of EEG-based MI systems [7].

A notable number of machine learning algorithms and feature extraction methods have been developed in order to improve the analysis of MI responses [8,9]. Another improving approach is to perform several training sessions in which participants learn how to modulate their sensorimotor rhythms appropriately, relying on the spatial specificity of MI-induced brain plasticity [10,11]. However, even after long training sessions, between 15% to 30% of users may not develop enough coordination skills [12,13], yielding an inadequate performance of most brain-computer interface (BCI) systems (termed the BCI inefficiency factor) and posing one of the biggest challenges in MI research.

One more enhancing strategy of learning is identifying the causes of variability and incorporating appropriate actions in order to compensate for the BCI inefficiency [14], for instance, by including a calibration module that works hand-in-hand with the training procedure to make learning algorithms adapt to user EEG patterns [15,16]. In this regard, the correlation between the neural activity features that are extracted in advance (electrophysiological indicators or predictor) with the MI onset responses instructed via sensory stimuli can be assessed to prescreen participants for the ability to learn regulation of brain activity (pre-training measures) or for the improvement of learning abilities (training phase) [17]. A systematic review of the predictors of neurofeedback training outcome is given in [18,19], concluding that the most promising predictor seems to be the (neurophysiological) baseline activity, which was derived from the parameter targeted by the training. In an attempt to anticipate the evoked MI responses, several pre-training electrophysiological indicators are reported, like functional connectivity of resting-state networks [20], α rhythm activity of eyes-open and eyes-closed resting-states [21], pre-cue EEG rhythms over different brain regions [22], and the power spectral density estimates of resting wakefulness (before the cue-onset of the conventional MI trial timing and resting state) [23,24]. Although this last predictor is one of the most used, its curve-fitting method depends heavily on various parameters that are difficult to determine, regardless of the resting data employed [25]. Other predictors are derived from measuring the change in electrophysiological properties across the training sessions [26,27]. Other predictors are derived from measuring the change in electrophysiological properties across the training sessions [26,27,28]. Thus, event-related Des/synchronization (ERD/ERS) is extracted in order to evaluate the (in)efficiency of MI training, which shows a distinct activation of the sensorimotor cortex region in response to imagery tasks [29]. Although visible ERD lateralization of evoked MI activity has been considered for predicting the user’s control ability from neurophysiological measures [30], the characterization of its topography and frequency specificity poses a challenging task because of the difficulty in accurately quantifying the trial-to-trial variability [31,32].

The linear correlation and regression models are used to explore or test the relationship between predictor and outcome measures, since they provide direct insight into the possible reasons for BCI control failures. However, the assumption of proportionality may be strong enough in real settings of MI tasks, resulting in scores with low values of significance. Instead, this task can be solved using linearizing models (like logistic regression [33]), which vary depending on the types and numbers of EEG indicators selected in each model [34]. Thus, related to motor evoked potential time series, nonlinear models (like random forests) can achieve significantly better prediction performance than a linear one (or logistic regression) [35]. In particular, machine learning analysis in nonlinear regression is extensively employed under two deep learning solutions [36,37]: (*i*) utilizing an ensemble of deep networks that suffer from larger computational complexity and (*ii*) transforming a single nonlinear regression hypothesis to a robust loss function that is jointly optimizable with the deep network usually in terms of the mean square error. However, the generalization ability is a major concern in developing deep regression models and computational complexity and hardware consumption [38].

Here, we develop a data-driven estimator, termed Deep Regression Network (DRN), which jointly extracts and performs the regression analysis to assess the efficiency of the individual brain networks in practicing MI tasks. Similar to the double-stage learning strategy for regression in [39], the proposed estimator initially learns a pool of deep patterns, extracted from the input data, in order to feed a neural regression model, allowing to infer the distinctiveness between subject assemblies having similar variability. The results, obtained on real-world MI data, prove that the DRN estimator fosters the ability of pre-training neural desynchronization and initial training synchronization to predict the bi-class accuracy response and, thus, providing a better understanding of the BCI-inefficiency of subjects.

The rest of the paper is organized, as follows: Section 2 briefly discusses the theoretical background of the model. Section 3 describes the experimental set-up, including the dataset used. Section 4 presents the assessment of Deep Regression Network performance, describes the results, and discusses the findings. Lastly, Section 5 concludes the paper.

## 2. Material & Methods

### 2.1. Electrophysiological Indicators in Mi Tasks

**Pre-training indicator of neural desynchronization:** for quantifying the potential for desynchronization at rest over the sensorimotor area, the spectral variability of a state of wakefulness conscious can be assessed by computing the difference between the EEG background activity (a fit of $f^{-1}$ noise spectrum) and the spectral content of those rhythms that are directly related to MI responses (i.e., μ and β). Thus, the pre-training neural predictor, noted as $\xi_{1}\in R^{+}$, is estimated while using the following fitting-curve based approach developed in [40]:

(1a) $$\begin{array}{cc} \xi_{1} & =\max_{\forall f\in f}\left\{s\left(f\right)-\varepsilon \left(f;\eta^{*},\kappa^{*}\right)\right\} \end{array}$$

(1b) $$\begin{array}{cc} \eta^{*},\kappa^{*} & =\{\arg\min_{m_{\Omega },\sigma_{\Omega },\eta ,k_{\Omega },\kappa }∥s(f)-\left(\sum_{\Omega =\mu ,\beta }k_{\Omega }N_{\Omega }\left(f;m_{\Omega },\sigma_{\Omega }\right)+\varepsilon \left(f;\eta ,\kappa \right)\right){∥}_{2}\} \end{array}$$

where s(f) is the positive semi-definite power spectral density (PSD) computed from an a priori given state of wakefulness, $N(f;m_{\Omega },\sigma_{\Omega })$ is a Gaussian function modeling each spectral peak of either sensorimotor rhythm Ω={μ,β}, widely reported for practicing MI tasks [41]; $\left\{k_{\Omega }\in R^{+}\right\}$ are the summation rhythm weights; $\left\{m_{\Omega }\in R^{+}\right\}$ and $\left\{\sigma_{\Omega }\in R^{+}\right\}$ are the spectral moments ruling the offset and scale of each fitting function, respectively; $\varepsilon \left(f;\eta ,\kappa \right)\kappa_{1}+\left(\kappa_{2}/f^{\eta }\right)$ is the hyperbolic fitting of noise with parameters $\left\{\kappa_{1}\in R^{+},\kappa_{2}\in R^{+}\right\},\eta \in R^{+}$. Notation ${∥\cdot ∥}_{p}$ stands for $\ell_{p}$-norm.

**Initial training indicator of Event-related De/Synchronization:** this time-locked change of ongoing EEG is a control-mechanism of the somatotopically organized areas of the primary motor cortex, which can be generated intentionally by mental imagery. For a measured EEG recording $x[x_{\Delta t}\in R]$, the estimation of ERD/ERS is performed, at specific sample Δt∈T, by squaring the samples and averaging over the EEG trial set to compute the variational percentage (decrease or increase) in EEG signal power regarding a given reference interval, as follows [42]:

(2) $$\begin{array}{cc} {\hat{\zeta }}_{\Delta t} & =\left(\zeta_{\Delta t}-\bar{\zeta }\right)/\bar{\zeta },\;s.t.:\mathrm{var}\left(\zeta_{\Delta t}\right)\gg \mathrm{var}\left(\bar{\zeta }\right), \end{array}$$

where $\zeta_{\Delta t}=E\left\{\right|x_{\Delta t,n}{|}^{2}\in x_{n}:\forall n\}$ is the power scatter averaged across the trial set, n∈N, and the trial power scatter $\bar{\zeta }=E\left\{\zeta_{\Delta t}:\forall \Delta t\in \Delta T_{1}\right\}$, with $\bar{\zeta }\in R$, is computed by averaging over the reference time interval $\Delta T_{1}\subset T$, being $T\in R^{+}$ the whole EEG recording length. The time-series of ERD/ERS is computed across the whole trial set by accurately ruling the trial power scatter $\bar{\zeta }\left(\cdot \right)$.

Intending to provide a scalar-valued assessment of the synchronization mechanism, the initial training indicator, which is noted as $\xi_{2}\in R^{+}$, is the distance measured between both labeled ERD/ERS time-series ($\lambda l,l^{\prime }$, denoting left-hand and right-hand tasks, respectively). ERD/ERS are extracted within each rhythm Ω at channel *c*, as below:

(3) $$\xi_{2}=\max_{\Omega ,c}\left\{\frac{∥\hat{\zeta }\left(\Omega ,c|l\right)-\hat{\zeta }\left(\Omega ,c|l^{\prime }\right){∥}_{2}^{2}}{∥\hat{\zeta }{\left(\Omega ,c|l\right)∥}_{2}{∥\hat{\zeta }\left(\Omega ,c|l^{\prime }\right)∥}_{2}}\right\}$$

where ζ(Ω,c|λ) is the estimated ERD/ERS at channel *c* and bandwidth Ω, selecting the baseline inverval as reference. The reported values of $\xi_{2}$ are computed using the maximization operator in Equation (3), relying on the fact that a single ERD/ERS time series may occur at different electrodes and bandwidths, being sufficient to provide an adequate neural response.

### 2.2. Regression Analysis between Classifier Performance and Electrophysiological Indicators

For evaluating the BCI efficiency, we employ a learning rule that estimates an unknown function $\theta :R^{M}\mapsto R$ from representative observations of an individual indicator (independent variable) $\xi \in R^{M}$, for which a multivariate model-free regression problem can be stated through by optimizing, across the subject set m∈M, the following framework:

(4) $$\min_{\pi }E\{∥\nu -\left(\theta \left\{\xi \left(x_{m}\right)|\pi \right\}+\epsilon \right){∥}_{p}:\forall m\in M,$$

where $\nu \in R^{M}$ is the response vector (dependent variable), $\epsilon \in R^{M}$ is the additive error term that is independent of ξ, and π is the unknown parameter vector that allows estimation of the function θ(;) that fits the data most closely in terms of a given $\ell_{p}$-norm distance.

Here, the framework in Equation (4) is further developed by a proposed data-driven estimator, termed Deep Regression Network (DRN), which jointly extracts and performs the regression analysis, as follows:

(5) $$\begin{array}{c} \min_{\pi }E\left\{∥\psi \left(V_{m}\right)-\left(\theta_{3}\circ \theta_{2}\circ \theta_{1}\left\{\xi \left(\varphi \left(x_{m}^{c}\right)\right):m,c\in M,C^{\prime }\right\}|\pi \right\}{)∥}_{1}:\forall m\in M\right\} \end{array}$$

where the initial hidden layer $\theta_{1}$ extracts through the function $\varphi (x^{c})$ as a set of salient patterns from all EEG recordings measured at every electrode $x^{c}$, $\theta_{2}$ is the fully-connected layer that maps the first-layer inputs into a high-dimensional space, generalizing the salient patterns sets over the considered channel configuration $C^{\prime }$ in order to assess the subject indicator $\xi^{*}$, $\theta_{3}$ is the output layer fed by the response set of individuals to perform the regression analysis by incorporating a linear activation function, $\psi (V_{m})$ is a functional that maps the scalar-valued response set $V_{m}$ assessed for each subject into a single value.

**Figure 1:**
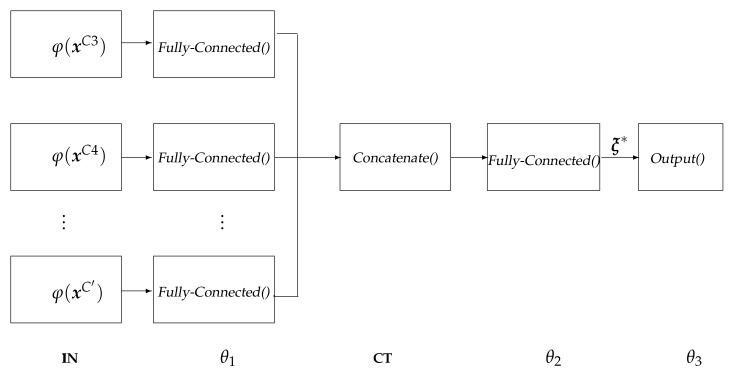
Proposed Deep Regression Network with three-layers architecture corresponding to the extraction of salient sensorimotor patterns, subject indicator computation, and the linear regression of performance responses on the assessed indicator vector.

Figure 1 sketches the proposed Deep Regression Network architecture that is based on the non-sequential Wide&Deep neural network to perform learning of deep patterns (using the deep path) under simple rules (through the short path) [43], implemented as below:

– **IN**: input layer that holds the extracted relevant patterns $\left\{\varphi \left(x_{m}^{c}\right):\forall c,m\right\}$.
– $\theta_{1}$: fully-Connected layer that is used for extracting robust and epileptic relevant patterns that are mapped into a high-dimensional latent space [44], holding h=⌜1.5size({φ(·)})⌝ neurons, being ⌜·⌝ the ceiling operator.
– **CT**: a concatenate layer that condenses the resulting feature sets of all electrodes into a single block, sizing $hC^{\prime }$.
– $\theta_{2}$: a fully-connected layer with size $⌜0.5hC^{\prime }⌝$ that is linked to each output-layer neuron.
– **$\theta_{3}$**: the one-neuron regression equipped with a linear activation function to predict the response.

Using the proposed Deep Regression Network framework, we extract the subject vector, which is noted as $\xi^{*}$, as an indicator of MI neural activity that is further correlated with the computed bi-class accuracy as a response variable. To this end, the parameters in Equation (5) are adjusted, as follows:

– The set of relevant patterns $\left\{\varphi \left(x_{m}^{c}\right)\right\}$ that holds elements extracted by the following statistical moments: mean, median, variance, minimal, and maximal values. For every subject, the moments are estimated over $x^{c}$ data using a short-time window lasting 1 s with a 50% overlap. All time-varying moments are concatenated to form a single set per channel.
– Both layers, $\theta_{1}$ and $\theta_{2}$, employ a hyperbolic tangent (*tanh*) as the activation function.
– During learning, *Adam algorithm* optimizer and *loss function* are used, measuring the Mean Absolute Error and fixing the learning rate to $10^{-3}$. In addition, the weight values (empirically set to $10^{-3}$) are regularized while using the Elastic Net regularization.
– The backpropagation algorithm solves the parameter set optimization of π with auto differentiation under a Wide Deep Neural Network framework that includes two hidden layers under elastic-net regularization.
– As the function mapping $\psi (V_{m})$, two operators over the response vectors are tested: (a) the mean accuracy (noted as mean) that is averaged across the extraction window lengths δτ and weighted by the subject variance performed at each window; (b) first PCA component of the accuracy vectors (noted as PCA${}_{1}$). The set $V_{m}$ is the subject accuracy values evaluated at four lengths of feature extraction δτ=[0.5,1.0,1.5,2.0] s, and performed over the whole trail MI data set, as explained before in Section 3.2.
– For evaluation purposes, we also contrast the DRN-based regression analysis with the case of avoiding the data-driven indicator extraction. Which is, the estimator in Equation (5) is directly fed by the scalar-valued neurophysiological indicators devised in Equations (1a) and (3), fixing each individual vector element of $\xi^{*}$ to $\xi^{*}=\xi_{1,2}$ and removing the concatenation layer **CT**.

## 3. Experimental Set-Up

Related to MI tasks, the methodology for evaluating the efficiency of neurophysiological indicators embraces the following stages: (*i*) extraction of a pre-training learning ability indicator, evaluating two scenarios of resting data for computation: (*a*) baseline inverval, $\Delta T_{1}$, lasting τ = 1.5 s; and (*b*) resting-state, lasting τ=55 s. (*ii*) Extraction of an initial training phase indicator from the Motor Imagery interval of the trial timing, (*iii*) regression and further clustering analysis between each electrophysiological indicator and the performance response of individuals. To this end, the accuracy classifier is estimated using the CSP-based features, maximizing the class variance to improve the system accuracy. Additionally, Spearman’s correlation coefficient is used to assess the effectiveness of each electrophysiological indicator considered in predicting the bi-class accuracy response.

In practice, extraction from fewer sensorimotor area is achieved in order to reduce the computational complexity without affecting the BCI system performance [45]. To this end, we select the EEG recordings measured over the sensorimotor area, evaluating two configurations of scalp positions: (*a*) narrow electrode arrangement (noted as 2Ch) that includes two channels ($C^{\prime }=2$): C3 (left motor cortical region) and C4 (right), (*b*) wide arrangement (6Ch) that holds six surrounding electrodes ($C^{\prime }=6$): C3 and P3 (left motor cortex), Cz and Pz (middle cortex), and C4 and P4 (right cortex).

### 3.1. MI Database Description and Pre-Processing

We explore the collection, publicly available at http://gigadb.org/dataset/100295, which holds EEG data obtained from fifty-two subjects using a 10-10 placement electrode system with C=64 channels. However, we only validate M=50 individuals, since two of them (#29 and #34) have less than 20 trials. Every channel x(c) lasting T=7 s was sampled at $F_{s}=512$ Hz. At the trial beginning, a fixation cross was presented on a black screen within a period that lasted 2 s. Subsequently, a cue instruction (related to either MI label λ=l or $\lambda =l^{\prime }$) appeared randomly on the screen for 3 s that inquired each subject to imagine moving his/her fingers, starting to form the index finger and proceeding to the little finger and touching each to their thumb. Afterward, a blank screen was shown at the beginning of a break period, lasting randomly between 4.1 and 4.8 s. For completing a single run, this procedure was repeated over 20 times and stopped at the end to fulfill a written cognitive questionnaire [46]. Every subject performed five or six runs. Additionally, a single-trial recording of resting-state lasting 60 s was collected from each subject.

**Figure 2:**
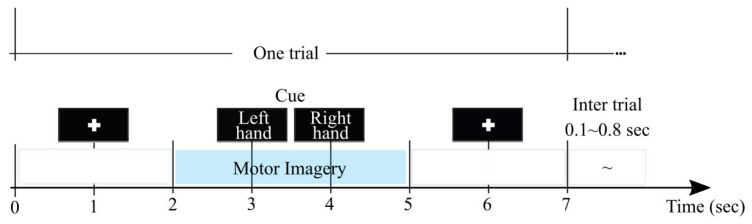
Block diagram and trial timing used to complete the MI database paradigm.

Every raw EEG channel was band-pass filtered within the frequency range f∈[4–40] Hz, covering both considered sensorimotor rhythms, μ and β. With the aim of providing a physiological interpretation of the implemented experimental paradigm, the MI dynamics pictured in Figure 2 are segmented. For purposes of evaluation, we employ the following two intervals of interest: $\Delta T_{1}=\;\left[0–2\right]$ s (termed baseline interval) and $\Delta T_{2}=\;\left[2.6-4.6\right]$ s (motor imagery interval). We only employ two intervals of interest during evaluation: $\Delta T_{1}$, which contains the baseline interval, and $\Delta T_{2}$, which reflects the most representative brain neural response. The length of either interval is selected to be comparable to the values that were reported for similar MI databases, like in [40].

For addressing the volume conduction problem, the indicators are assessed after performing the Laplacian filter over the input EGG data to improve the spatial resolution of EEG recordings This filtering procedure was carried out using *Biosig Toolbox*, freely available at http://biosig.sourceforge.net, avoiding the influence of noise coming from neighboring channels [47]. Of note, the first five seconds are removed from resting data because of measured variations [48].

### 3.2. Bi-Class Accuracy Estimation as a Response Variable

We perform the individual accuracy in distinguishing either MI class as the performance response in order to validate the proposed data-driven estimator approach. The classifier accuracy is computed using the sliding short-time feature set extracted by the algorithm of Common Spatial Patterns (CSP), fixing the surrogate space variance to the first three eigenvectors by class, as carried out in [49]. It is worth noting that the short-time window must be adjusted for extracting the subject EEG dynamics over time accurately. To reflect this influence, we test four different lengths of the sliding window: δτ=[0.5,1.0,1.5,2.0] s, having an overlap of 50%.

**Figure 3:**
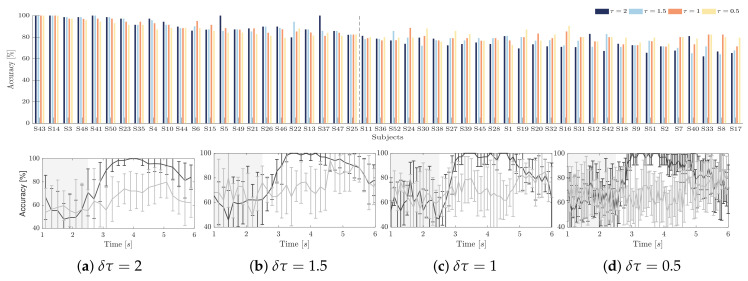
Individual accuracy in distinguishing either MI class performed by the CSP-based time-frequency feature set, using different window lengths: **–** δτ=2, **–** δτ=1.5, **–** δτ=1, **–** δτ=0.5. Bottom row: Accuracy for the trial timing using different windows S14 (marked with color **–**) and S17(**–**).

The top row in Figure 3 displays the classification accuracy achieved by each individual at different δτ, employing the Linear Discriminant Analysis algorithm and applying the regularized selection strategy over the extracted CSP feature set together with a 10×10-fold cross-validation scheme, as carried out in [50]. For purposes of interpretation, all of the individuals are ranked in decreasing order according to the achieved CSP-based accuracy, showing that the less the classifier performance, the higher the dispersion between accuracy estimates extracted at different window lengths δτ. However, the subjects performing the best have better accuracy at length δτ=2, while the worst individuals achieve better at the shorter window δτ=0.5, which means that the dynamics of neural responses may cluster into different groups in terms of the utilized extraction length δτ.

As an illustration, the bottom row in Figure 3 draws the time-varying classification accuracy achieved by two representative subjects: the individual labeled as S14 that reaches very high scores across the whole MI interval and the subject S17 that presents the lowest distinguishing ability, performing the highest accuracy unusually late (after the expected $\Delta T_{2}$ interval).

## 4. Results and Discussion

### 4.1. Computation of Pre-Training Desynchronization Indicator

For extracting the PSD-fitting values in Equation (1a), the power spectral density s(f) of each Laplacian-filtered channel, $\left\{x^{c}\right\}$, is computed through the nonparametric Welch’s method. To this, we use a set of smooth-time sliding windows of length 1 s, fixing an overlap of 50% in order to overcome the non-stationary nature of EEG data. Further, we perform a single estimate of $\xi_{1}$ as the mean value averaged across the tested scalp electrode configuration.

**Figure 4:**
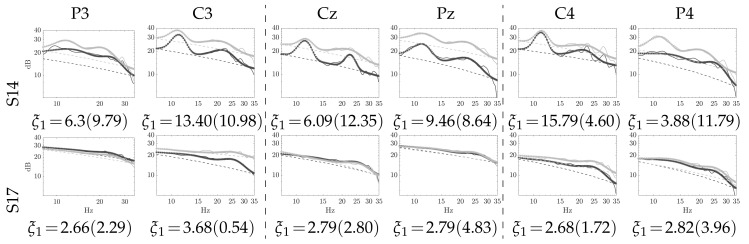
Examples of pre-training power spectral density (PSD)-fitting computed within resting data: baseline inverval (black line) and resting (gray line). Values of $\xi_{1}$ are reported for the sensorimotor area of baseline inverval and (resting) states.

Figure 4 depicts the curve-fitting model obtained, respectively, by the baseline interval (outlined in black color) and resting-state (gray color). The PSD estimate is drawn by a continuous line, the curve-fitting–by an asterisk line, and the hyperbolic fitting of noise–by a dashed line. In the case of subject # 14 reaching high accuracy, the top row presents the performed curve-fitting with a high indicator value, showing a big match between the modeled and PSD estimated from the resting-state in each one of the six considered channels. As expected, the spatial configuration 2Ch provides the best values of $\xi_{1}$, which are large enough when compared with the remaining channels. On the contrary, the subject # 17 with a very low accuracy performs a small indicator because of a poor fitting agreement (see the bottom row), also having no distinguishable activity at μ and β rhythms, regardless of the channel. The values of curve-fitting adjustment are shown beneath the plots, resulting in very close estimates for the pre-training desynchronization indicator despite the resting data extraction interval.

**Figure 5:**
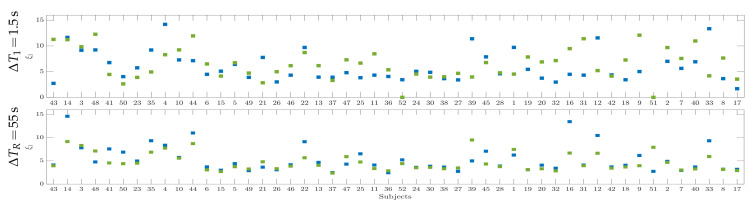
Pre-training desynchronization indicator $\xi_{1}$ computed for baseline inverval (top plot) and resting data (bottom row) while using either electrode arrangement: 2Ch (blue color) and 6Ch (green color). Individuals are ranked according with the achieved accuracy response.

Figure 5 displays the indicator that was calculated by Equation (1a) according to the achieved CSP-based accuracy that is ranked in decreasing order. As seen in the top row, the baseline inverval estimates extracted from 2Ch configuration (colored with blue squares) have a behavior that is comparable to the values that were recomputed by expanding to 6Ch the number of MI channels (green squares). A similar situation holds for the resting state indicator computed, as observed in the bottom row. It is worth noting that, although there is a high resemblance between both individual assessments (close to 50%), either calculated version of $\xi_{1}$ barely follows the ranked accuracy sequence of individuals.

### 4.2. Initial Training Synchronization Assessment

Here, we extract the ERD/ERS dynamics over the entire filtered trial matrix, fixing the time window to the sample rate (0.004 s). Additionally, the reference interval is fixed to the range 0.5–1.5 s while using the significance value of 1% in *z*-score approach, as performed in [51].

**Figure 6:**
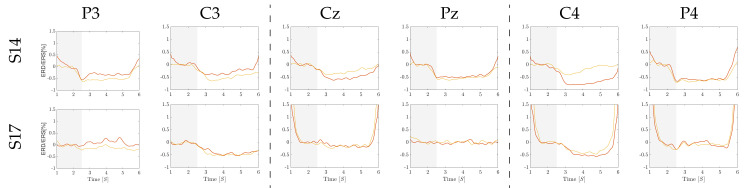
Exemplary ERD/ERS time-courses performed by subjects S17 and S14 for left-hand class (colored in red line) and right-hand class (in yellow) at the evaluated scalp electrodes, while using the back resting state (shadowed area) as the reference segment.

Figure 6 displays the individual pattern changes extracted from the electrode arrangement Ch6, holding the cue onset interval (shadowed area) and the MI segment $\Delta T_{2}$. As seen, the induced synchronization mechanisms are represented through the increase or decrease of energy at the post-stimulus period. For illustration purposes, the corresponding time series are presented for a couple of representative subjects: #14 that performs high accuracy and #17, achieving a low accuracy. The former individual provides distinctive modulation amplitudes all over the sensorimotor area, while the latter subject presents a weak synchronization behavior, as observed in the top row.

**Figure 7:**
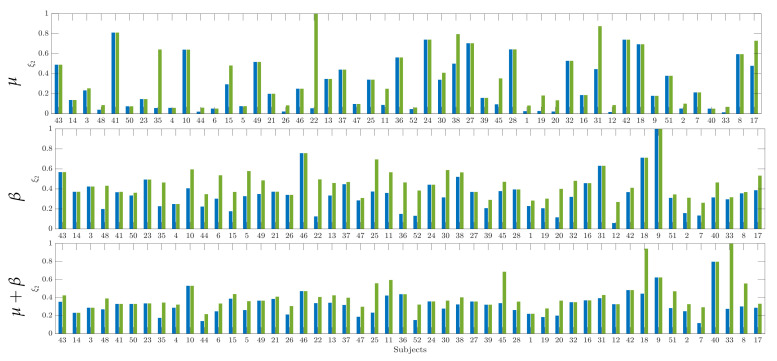
Individual values of initial training synchronization $\xi_{2}$ computed within subband combinations: μ,β,μ+β.

Further, Figure 7 displays the assessments of individual synchronization that are computed while using the labeled-related distance in Equation (3) within the sensorimotor rhythms, for which the electrical brain activity prompted by motor tasks is frequently associated. The computed values of initial training synchronization $\xi_{2}$ hardly follow the accuracy sequence of individuals, as observed in the previous indicator.

**Figure 8:**
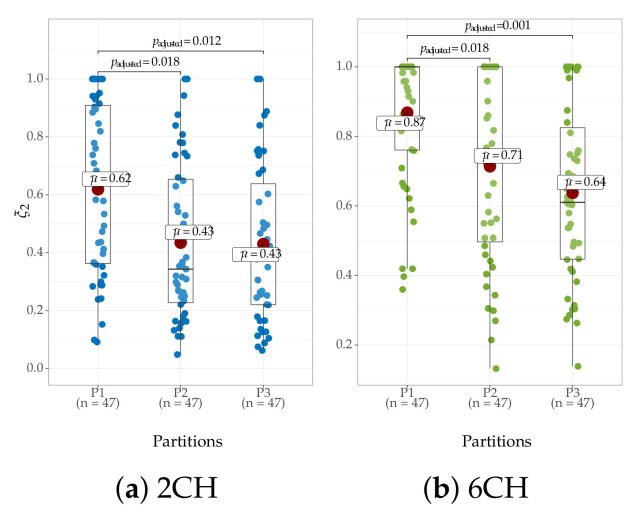
Differences in initial training synchronization $\xi_{2}$ performed at each trial partition during the training sessions.

One more aspect to consider is the indicator’s capacity to characterize the training session’s synchronization mechanism. To this end, we extract $\xi_{2}$ while using a sequence of 30 trials ordered in time. Fixing a significance value of 5%, Figure 8 displays the results of the Wilcoxon signed-rank test, revealing that the first 30 trials are different from the second run. Likewise, the second run differs from the last one (only three runs are considered, since not all subjects have the same number of trials). Moreover, the mean value of $\xi_{2}$ decreases over the runs, which suggests that the synchronization mechanism can be evaluated as the training sessions increases in number. Overall, these outcomes in Figure 8 agree to the results in [52], evidencing the difficulty of quantifying a significant change in ERD/ERS across the training sessions, even for either channel C3 or C4.

### 4.3. Drn-Based Indicator Extraction and Regression

Aiming at assessing the effectiveness of the pre-training desynchronization indicator $\xi_{1}$, Table 1 displays Spearman’s correlation coefficient, *r* ∈ ℝ, which is reported under two different regression assumptions: linear (noted as LC) and linearized (DRN). In the case of extracting $\xi_{1}$ by Equation (1a) from the baseline inverval, the linear correlates with the responses yield a minimal value of *r*, regardless of the associated accuracy response. The efficiency for predicting the subject accuracy remains not significant (r<0.23), even though the expanded electrode arrangement increases the Spearman coefficient a little. Further, the values of *r* are performed through the linearizing DRN estimator while using the same scalar-valued PSD-fitting indicator set (noted as DRN ${\tilde{\xi }}^{*}=\xi_{1}$), which is obtained by concatenating all of the trials before carrying out the short-time vector extraction, as implemented in [40]. As a result, the correlation with the MI performance raises to r<0.37, but this indicator poses still not meaningful for prediction. Lastly, the use of the DRN framework for joint indicator extraction and regression (noted as DRN $\xi^{*}$) leads to a notable increase of the Spearman coefficient up to r<0.88, allowing for an adequate predictive interpretation of the data-driven pre-training desynchronization indicator.

When extracting $\xi_{1}$ by Equation (1a) from a single resting-state record, the linear assumption increases almost by two the values of *r* as compared to the previous baseline inverval extraction. This result may point out that the resting-state data enable a more confident estimation of the desynchronization indicator. Nonetheless, for these scalar-valued estimates, the DRN estimator cannot further improve their predictive ability with the accuracy responses (r<0.40). However, the joint model of DRN-based indicator extraction and regression leads to a definite rise in the correlation coefficient, outperforming all of the tested scenarios of resting data (r<0.93).

The linear correlation values of (r<0.39) performed by the initial training synchronization $\xi_{2}$ are comparable to the ones of $\xi_{1}$, including both evaluated rhythm bandwidths μ+β and the wide electrode arrangement, as presented in Table 2. By feeding the DRN estimator with the scalar-valued $\xi_{2}$ (noted as DRN ${\tilde{\xi }}^{*}=\xi_{2}$), similar low significant correlation values are obtained, regardless of the evaluated rhythms. The fact that the proposed DRN estimator is not benefiting from a scalar-valued indicator set implies that involved Wide&Deep neural network demands a higher volume of information from predictors to perform learning of deep patterns.

On the other hand, the characterization of evoked MI activity poses a challenging task, because of the difficulty in quantifying the trial-to-trial variability accurately, increasing the complexity in assessing the distance $\xi_{2}$ between both labeled ERD/ERS time-series by Equation (3). It should be noted that the indicators perform the best linear estimates of *r* at a distinct window length (δτ=0.5 by $\xi_{1}$ while δτ=0.5 by $\xi_{2}$), which means that this extraction parameter must be tuned differently for each indicator.

Once again, the DRN framework of joint indicator extraction and regression (DRN $\xi^{*}$) enables an increase of the Spearman coefficient up to r<0.89, concatenating both labeled ERD/ERS time series at the estimator input. Therefore, for increasing the predictive interpretation of either considered electrophysiological indicator, the proposed DRN framework should incorporate the joint extraction and regression procedures, intending to extract more distinguishing information between subjects from the indicators.

### 4.4. Clustering of Subject-Level Efficiency

Here, we assume the rationale by which the higher the accuracy in distinguishing between MI tasks, the more efficient the individual brain network. Therefore, the sets of the extracted indicator values, together with the accuracy series, are employed to infer the distinctiveness between the subject assemblies, each having a similar variability level.

In the beginning, we determine the number of partitions considering the intra and inter-subject variability of responses as an important factor affecting the regression analysis that was conducted by Equation (5). Thus, an adequate group number is found to be three, which we estimate through the k-means algorithm fed by the four accuracy sets accounting for the performance variability, because of the extraction window length, δτ (see Figure 3), and introducing the cluster inertia and the Silhouette score to minimize the objective function.

**Figure 9:**
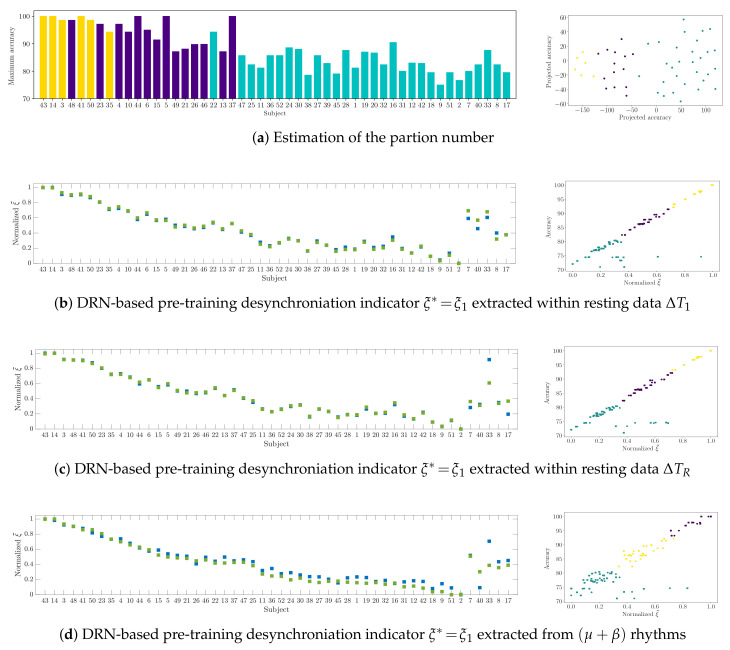
Extracted assessments using the proposed DRN estimator (left-side column) and performed clustering of subjects (right-side column).

The top row in Figure 9 displays the maximal accuracy that was performed by each subject within the extraction window set and his assigned group (left plot). The corresponding right plot depicts the resulting cluster by the colored dots into the following three partitions of individuals:

*(i)*  A group that holds the individuals performing the best accuracy with very low variability (yellow color).
*(ii)*  A group that contains the subjects that reach important values of accuracy, but performing with some fluctuations.
*(iii)*  A group with modest accuracy performed with high unevenness.

In the following, each group is assumed to have distinguishable skills in practicing Motor Imagery tasks.

The rows (b)–(d) in Figure 9 present the indicators that were extracted by the proposed DRN in Equation (5) that perform the best Spearman correlation *r*, meaning that they provide a high ability to predict the bi-class accuracy response. It is worth noting the high linearity between each indicator and the performance response set ranked in decreasing order, as displayed in the left column. The right column depicts the three subject partitions that were accomplished by the DRN extracted indicators, which are evidently separated, regardless of the involved indicator. Furthermore, the similarities between 2Ch (colored with blue squares) and 6Ch (green squares) arrangements are not noticeable, meaning that the clustering is scarcely affected by the fluctuations of neural activity coming from neighboring electrodes.

Nonetheless, as seen at the end of the left-side plots, several subjects (namely, #7, #40, #33, #8, and #17) do not follow the trend, and they are out of the regression (right plots), which implies that the DNR framework is not able to linearize the indicators extracted from this group of subjects. Besides their lowest performed bi-class accuracy, the main reason accounting for this discrepancy is the implied variability in their response that exceeds the performed values by the remaining subject set, as explained before in Figure 3a. In fact, the outlier subject set’s classification performance increases atypically at the end of the MI interval, so that some subjects do not provide distinguishable activity between μ and β rhythms. This issue seems to be relevant, since it proves that, along with the measured indicator variability, the response behavior also changes influence the resulting data-driven regression analysis. Consequently, the number of subject partitions is increased by one, and the appearing fourth group contains the outlier subject set for which the DRN estimator cannot infer any predictive ability because of their intra-subject variability.

**Figure 10:**
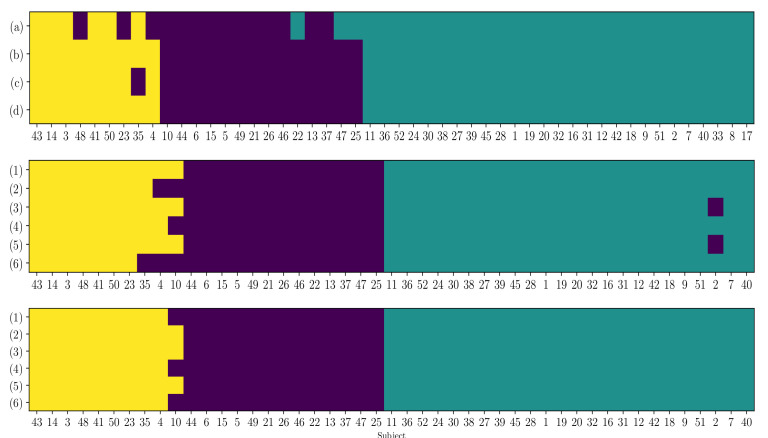
Clustering of individuals according to the DRN-based indicator extraction and regression. In first row, notations (a), (b), (c), and (d) stand for the corresponding items in Figure 9. The last two rows show the cluster of the DRN-based indicator $\xi^{*}=\xi_{2}$ extracted from (μ+β) rhythms, removing 10 trials consecutively in six runs with 2CH and 6CH electrode configuration, respectively.

Another concern is how few subjects can exchange the assigned clusters when accounting for each extracted indicator’s influence. To illustrate this fact, in Figure 10 we display the matrix that spans the cells colored according to the individual group assigned by the DRN-based estimator. The top row shows that the just a couple of subjects downgrades from the group I to II, when utilizing the extracted by DRN-based indicator assessments (see the pictured sets of (b)–(d) in Figure 9). It is worth noting that either electrode arrangement performs the same clustering if it involves the entire trail set of EEG data.

## 5. Concluding Remarks

To provide a better understanding of the BCI-inefficiency, we develop a data-driven estimator, termed Deep Regression Network (DRN), which jointly extracts and performs the regression analysis to assess the efficiency the individual brain networks in practicing MI tasks. To deal with the high inter- and intra-subject variability of elicited neural activity, the estimator performs learning of deep patterns, allowing to infer the distinctiveness between subject assemblies having similar variability. The results, which were obtained on real-world MI data, prove that the DRN estimator fosters the ability of the pre-training neural desynchronization and initial training synchronization to predict the bi-class accuracy response and, thus, providing a better understanding of the user’s intent of action upon imagination tasks. The regression-based evaluation of the tested neurophysiological indicators for predicting the subject’s ability to practice motor imagery tasks implies the following aspects:

*Electrophysiological indicators in evaluation efficiency*. We appraise the ability of pre-training neural desynchronization to predict the system response, showing that the computation by the baseline PSD-fitting may result in low significant correlates to the bi-classification accuracy (r<0.23), at least, if performing extraction from the back-resting state. By extracting from resting-state data, the correlation with the MI performance raises to r<0.37, remaining still not meaningful for prediction. Besides, the initial training synchronization indicator is assessed while using a proposed distance between both labeled Event-related De/Synchronization time-series that hardly follows the accuracy, sequence of individuals, resulting in low significant correlation values, regardless of the evaluated rhythms. However, other approaches of ERD/ERS calculation are to be evaluated, like event-related spectral perturbation technique [53].

*Classifier accuracy as a response variable*. In order to assess the efficiency of individual brain networks, the accuracy in distinguishing between MI tasks is widely employed, which is frequently computed while using the sliding short-time feature set extracted by the algorithm of Common Spatial Patterns. However, to deal with the intra inter-subject variability, the short-time length must be adjusted for each subject properly (see Figure 3). Furthermore, the individuals performing the worst are more susceptible to this choice, degrading the regression analysis highly. As a result, either indicator’s predictive ability depends differently on this extracting parameter, at least, using linear regression (see Table 1 and Table 2). This result may lead to a restriction when gathering several electrophysiological indicators into a common regression framework to improve efficiency evaluation of subjects.

*Joint model of indicator extraction and regression analysis*. For increasing the predictive interpretation of either considered electrophysiological indicator, we develop a Deep Regression Network framework that, first, extracts from neural activity indicators the most salient patterns that allow evaluating the BCI inefficiency, and then performs linearization of the indicator assessments towards the accuracy response. As a result, there is high linearity between the extracted sets for either indicator and the ranked performance response values of subjects. To include the accuracy variability because of window extraction, we test the mean accuracy weighted across the subject variance and the first PCA eigenvalue of the accuracy vectors, both performing similarly and outperforming notably the results that were obtained by each particular window length. Nonetheless, the proposed DRN estimator does not benefit from scalar-valued indicator sets, since the included Wide&Deep neural network demands a larger amount of information from predictors to perform learning of deep patterns.

One more aspect to remark is that the developed prediction model is subject-dependent and has to be validated with trial sets acquired under similar conditions from a representative number of individuals. As a rule, publicly available motor imagery databases are small, unusually exceeding several dozens because of their associated cost of implementation. We also need to validate the resting-state data that are less present in MI collections, since their capture demands a different paradigm, increasing the acquisition complexity. Here, we use the leave-one-out-cross validation strategy (LOO) to reduce the variability derived by splitting into two groups the validating data (training and test), enhancing the generalizing ability of the developed predictor and the model reproducibility, even under such an amount of examined individuals, aiming to understand why some subject groups show different performances in the same system.

*Cluster of subject efficiency*. The extracted indicator assessments, together with the accuracy series, are employed to infer the distinctiveness between the subject groups with a comparable variability level, that is, having similar skills in practicing MI tasks. As a result, the DRN estimator provides three subject partitions with the predictive ability regardless of the involved indicator and barely affected by the fluctuations of neural activity coming from neighboring electrodes. One more group with nonpredictive ability is obtained that holds the subjects with the lowest and most variable estimates of accuracy. The DNR framework is not able to linearize this group, which confirms that the changes in the response behavior also influence the resulting data-driven regression analysis.

Nonetheless, some issues remain to enhance the BCI-inefficiency evaluation through the developed data-drive DRN estimator. Firstly, the extraction of indicators should be improved; for instance, the assessment of the initial training synchronization must be performed using more elaborate labeled-based distances. Generally, the $\ell_{2}$ loss function tends to limit the generalization ability due to its susceptibility to outliers. Instead, using the combined $\ell_{2,1}$-norm concept loss (or even $\ell_{\infty }$-norm), the curve-fitting indicator in Equation (1a) can be improved. Further, the DRN framework should be enhanced in order to include the joint extraction of several indicators, taking into account the differences in the de/synchronization mechanism between both brain hemispheres. Additionally, there is a need to develop a more powerful mapping function to include the system response’s stochastic behavior. Another aspect of improving is the Deep Network architecture to enhance the interpretation of spatial brain neural patterns that mainly contribute to evaluating indicators’ efficiency in practicing MI tasks.

As future work, we plan to validate the proposal on a database with more subjects to obtain more robust evidence of the presented findings. Additionally, a broader class of MI dynamics is to be considered together with subjective scores of perception assessments, aiming to understand why some subject groups show different performances in the same system. 

## Figures and Tables

**Table 1:** Biserial Spearman correlation coefficient quantified between the $\xi_{1}$ indicator, extracted within different scenarios of resting data, and the accuracy response, estimated at each window length of δτ. Notations LC, DRN, and LOO stand for Linear Correlation [40], Deep Regression Network, and leave-one-out-cross validation strategy, respectively. The best value per row is marked in bold.

*Resting Data*	*Electrode*	δτ [*s*]				ψ(·)	
	*Configuration*	0.5	1.0	1.5	2.0	*Mean*	PCA${}_{1}$
Baseline inverval	2Ch(LC)	0.15	0.15	**0.17**	0.16	0.13	0.15
	6Ch(LC)	0.07	0.04	0.11	**0.13**	0.05	0.07
	2Ch(DRN $\xi^{*}=\xi_{1}$)	0.15	0.16	**0.18**	0.16	0.14	0.15
	6Ch(DRN $\xi^{*}=\xi_{1}$)	0.07	0.04	0.12	**0.14**	0.06	0.08
	2Ch(DRN $\xi^{*}$)	0.86	0.85	0.96	**0.97**	0.83	0.87
	2Ch(DRN $\xi^{*}$) LOO	0.76	0.79	0.82	0.80	0.78	**0.86**
	6Ch(DRN $\xi^{*}$)	0.92	0.86	0.95	**0.97**	0.83	0.88
	6Ch(DRN $\xi^{*}$) LOO	0.83	0.87	0.85	0.87	0.89	**0.91**
Resting-state	2Ch(LC)	0.30	0.31	0.31	0.27	0.29	**0.31**
	6Ch(LC)	0.25	0.31	0.26	0.26	**0.28**	**0.28**
	2Ch(DRN $\xi^{*}=\xi_{1}$)	0.31	0.31	0.31	0.28	0.30	**0.32**
	6Ch(DRN $\xi^{*}=\xi_{1}$)	0.25	**0.31**	0.26	0.27	0.30	0.30
	2Ch(DRN $\xi^{*}$)	0.79	0.80	0.92	**0.94**	0.78	0.82
	2Ch(DRN $\xi^{*}$) LOO	0.85	**0.87**	0.83	0.82	0.79	0.84
	6Ch(DRN $\xi^{*}$)	0.86	0.77	0.91	**0.93**	0.75	0.80
	6Ch(DRN $\xi^{*}$) LOO	0.85	0.83	**0.88**	0.86	0.80	0.77

**Table 2:** Computed values of *r* for the indicator of initial training synchronization within the evaluated rhythm bandwidths: μ,β,μ+β. Notations LC, DRN, and LOO stand for Linear Correlation [40], Deep Regression Network, and leave-one-out-cross validation strategy, respectively. The best value per row is marked in bold.

*Rhythm*	*Electrode*	τ [*s*]				ψ(·)	
*Subband*	*Configuration*	0.5	1.0	1.5	2.0	*Mean*	PCA${}_{1}$
μ	2Ch(LC)	**0.12**	0.064	0.04	0.003	0.6	0.05
	6Ch(LC)	**0.23**	0.08	0.10	0.04	0.11	0.11
	2Ch(DRN $\xi^{*}=\xi_{2}$)	0.13	0.064	0.13	**0.17**	0.06	0.17
	6Ch(DRN $\xi^{*}=\xi_{2}$)	**0.23**	0.12	0.10	0.04	0.11	0.11
β	2Ch(LC)	**0.11**	0.06	0.08	0.02	0.07	0.06
	6Ch(LC)	**0.14**	0.04	0.006	0.016	0.11	0.07
	2Ch(DRN $\xi^{*}=\xi_{2}$)	0.16	0.15	0.20	**0.23**	0.16	0.20
	6Ch(DRN $\xi^{*}=\xi_{2}$)	0.19	0.05	0.23	**0.25**	0.21	0.20
μ+β	2Ch(LC)	**0.06**	0.05	0.05	0.01	0.04	0.04
	6Ch(LC)	**0.11**	0.07	0.03	0.04	**0.11**	0.08
	2Ch(DRN $\xi^{*}\xi_{2}$)	0.08	0.06	0.10	**0.18**	0.11	0.09
	6Ch(DRN $\xi^{*}\xi_{2}$)	0.11	0.11	0.19	**0.21**	0.15	**0.21**
	2Ch(DRN $\xi^{*}$)	0.84	0.80	**0.94**	0.91	0.78	0.83
	2Ch(DRN $\xi^{*}$) LOO	0.15	0.17	**0.24**	0.19	0.18	0.21
	6Ch(DRN $\xi^{*}$)	0.87	0.77	0.93	**0.95**	0.82	0.82
	6Ch(DRN $\xi^{*}$) LOO	0.20	**0.44**	0.40	0.28	0.26	0.40
